# Efficacy of tyrosine kinase inhibitors in patients with non-small-cell lung cancer with performance status 4: a case series and review of the literature

**DOI:** 10.1186/s13256-023-04145-z

**Published:** 2023-09-28

**Authors:** Akinori Sasaki, Yutaro Fujimoto, Takashi Inada, Azusa Ishizuka, Jun Ehara, Shin Ogita, Yasuhiro Norisue

**Affiliations:** 1 Department of Oncology, Tokyo Bay Urayasu Ichikawa Medical Center, 3-4-32 Toudaijima, Urayasu, Chiba 279-0001 Japan; 2 Department of Pulmonology, Tokyo Bay Urayasu Ichikawa Medical Center, 3-4-32 Toudaijima, Urayasu, Chiba 279-0001 Japan; 3 Department of Oncology, St. Luke’s International Hospital, 9-1 Akashi-Cho, Chuo, Tokyo 104-8560 Japan

**Keywords:** Best supportive care, Non-small-cell lung cancer, Older patients, Performance status 4, Tyrosine kinase inhibitor

## Abstract

**Background:**

Current guidelines for non-small-cell lung cancer (NSCLC) recommend that each tyrosine kinase inhibitor (TKI) is indicated even for driver mutation-positive patients with a poor performance status (PS). In previous studies, most patients had a PS of 2–3, but those with a PS of 4 were very few. Therefore, the efficacy of TKIs in patients with NSCLC with a PS of 4 remains unclear.

**Case presentation:**

We retrospectively reviewed the clinical records of four patients with NSCLC with PS 4 treated with TKIs: an 89-year-old Japanese woman (Case 1), a 80-year-old Japanese woman (Case 2), an 50-year-old Japanese man (Case 3), and a 81-year-old Japanese woman (Case 4). Genetic alterations were epidermal growth factor receptor (*EGFR*), *MET* exon 14 skipping, *BRAF*V600E, and *ROS1* proto-oncogene receptor tyrosine kinase (*ROS1*). One case with *ROS1* fusion showed a significant response with the recovery of PS. However, in the remaining three cases (i.e., *EGFR*, *MET* exon 14 skipping, and *BRAF*V600E mutations), patients died despite the administration of TKIs. These three patients had to be hospitalized at the end of their life to receive treatment.

**Conclusions:**

This is the first case series to summarize the efficacy of TKIs in patients with NSCLC with a PS of 4. Additionally, this case series poses a question concerning the indication of TKIs for older patients with a PS of 4.

## Background

Lung cancer is the most common type of cancer and the leading cause of cancer-related mortality worldwide [1]. Non-small-cell lung cancer (NSCLC) accounts for > 70% of all lung cancers and is usually diagnosed at an advanced stage [2].

Generally, driver alterations and expression of programmed death ligand-1 are assessed before chemotherapy initiation for advanced or metastatic NSCLC. Important driver alterations for the medical treatment of advanced or metastatic NSCLC include epidermal growth factor receptor (*EGFR*), anaplastic lymphoma kinase (*ALK*), ROS1 proto-oncogene receptor tyrosine kinase (*ROS1*), *BRAF*V600E, *MET* exon 14 skipping, and *RET*. Tyrosine kinase inhibitors (TKIs) have a higher tumor response rate and shorter response time than cytotoxic chemotherapy for patients with driver alteration-positive NSCLC [3–8]. Therefore, TKIs are the standard treatment for such patients having a good performance status (PS).

Additionally, previous studies have reported that several TKIs have shown significant efficacy in patients with extremely poor PS [9–11]. These studies demonstrated an improved PS score, progression-free survival, and overall response rate in most patients. The Japanese and National Comprehensive Cancer Network (NCCN) and European Society for Medical Oncology (ESMO) guidelines recommend that each TKI be indicated even for diverse mutation-positive patients with poor PS [12–14]. However, most patients had a PS of 2–3, but only a few had a PS of 4 in previous studies. Therefore, the efficacy of TKIs in patients with NSCLC with a PS of 4 remains unclear. Moreover, limited clinical information is available regarding older patients with poor PS.

This case series describes our experience in treating patients with a PS of 4 with TKIs. Three out of four patients were aged ≥ 80 years, which is classified as “oldest old.” In this article, we discuss whether treatment with TKIs is effective for older patients with a PS of 4.

## Case presentation

### Case 1

An 89-year-old Japanese woman presented with symptoms of chronic cough and weight loss and was diagnosed with NSCLC in February 2020. Computed tomography (CT) findings showed primary lung cancer in the left hilar area, mediastinal lymph node metastasis, and bilateral pleural effusions. She had never smoked, and she had a medical history of angina. Pleural fluid cytology was positive for malignant cells. Following a cell block from the malignant pleural effusion, the adenocarcinoma was pathologically confirmed. The patient’s initial PS score was 2. She was admitted to our hospital with dyspnea due to bilateral pleural effusions and oxygen therapy was initiated.

**Fig. 1:**
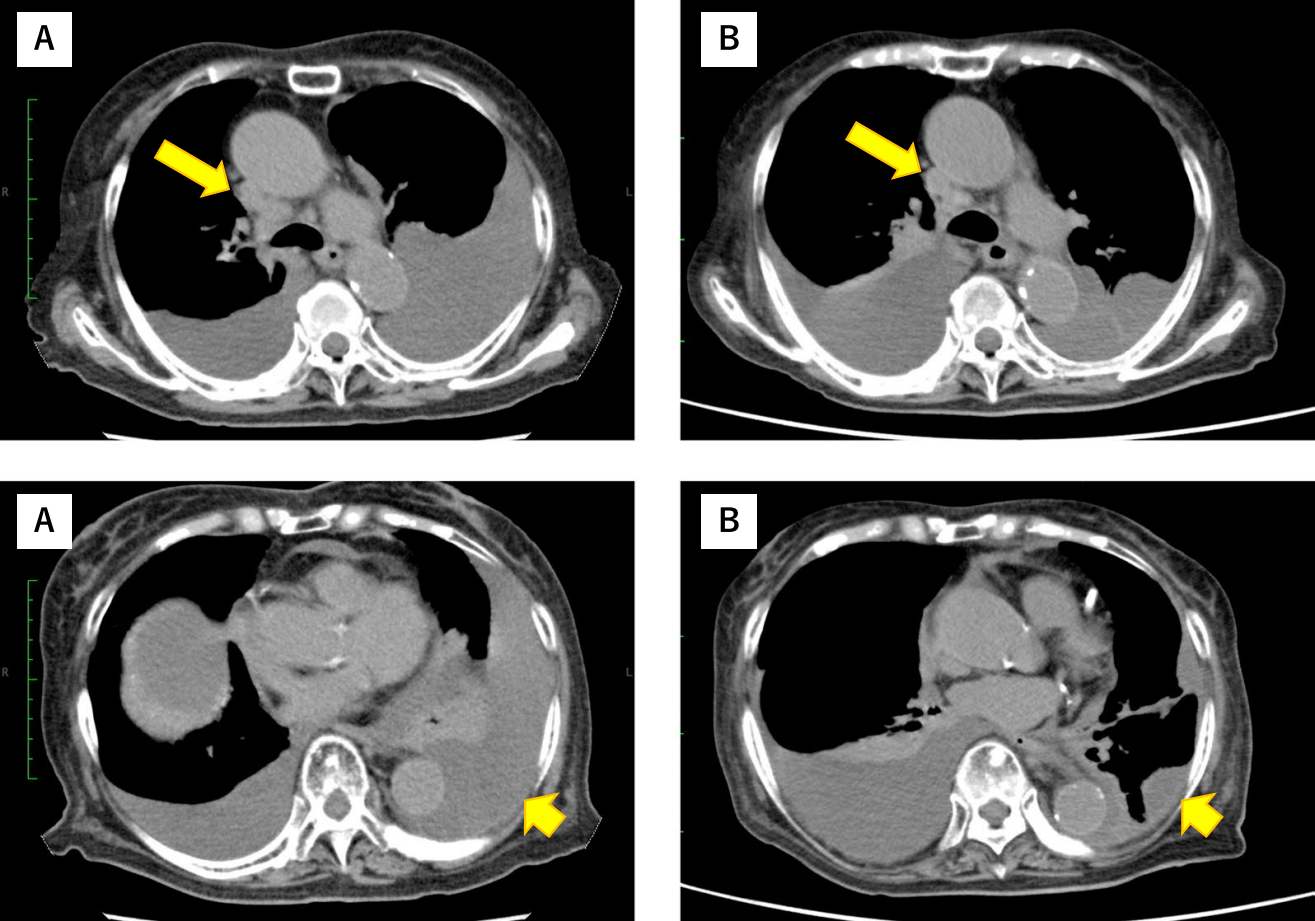
Chest computed tomography images before and after administering osimertinib. **a** Mediastinal lymph node metastasis and pleural effusions before treatment. **b** Slight tumor shrinkage in mediastinal lymph node metastasis (long arrow) and decrease in left pleural effusion (short arrow)

After hospital admission, she underwent a bronchoscopy for tissue biopsy. Subsequently, she started receiving treatment for hypoxemia until the pathology results, including those for driver mutations, became available. The *EGFR* L858R mutation was confirmed in tumor specimens on day 20. However, her general condition deteriorated owing to prolonged immobility and the progression of pleural effusion, resulting in a PS score of 4. Following a discussion, the patient decided to receive TKI treatment. Thus, osimertinib was administered at a standard dose of 80 mg once daily. After several days of osimertinib administration, the patient stopped receiving treatment because of grade 2 vomiting and diarrhea. Chest CT images at that time showed a decrease in malignant pleural effusions and slight shrinkage of the mediastinal lymph node metastasis (Fig. 1). However, her PS was not improved; thus, she opted to discontinue osimertinib because of adverse events. The patient died at the hospital on day 35.

### Case 2

An 80-year-old Japanese woman presented with symptoms of fatigue and dyspnea and was diagnosed with stage IV NSCLC in August 2020. CT findings showed primary lung cancer in the left lower lobe and bilateral cervical and mediastinal lymph node metastases. She had never smoked and had a history of diabetes mellitus and hypertension. Following transbronchial biopsy, adenocarcinoma was pathologically confirmed. She presented to the hospital with dyspnea and was subsequently diagnosed with superior vena cava syndrome, which was treated by endovenous stent placement. She underwent endotracheal intubation because of worsening respiratory status and was admitted to the intensive care unit.

**Fig. 2:**
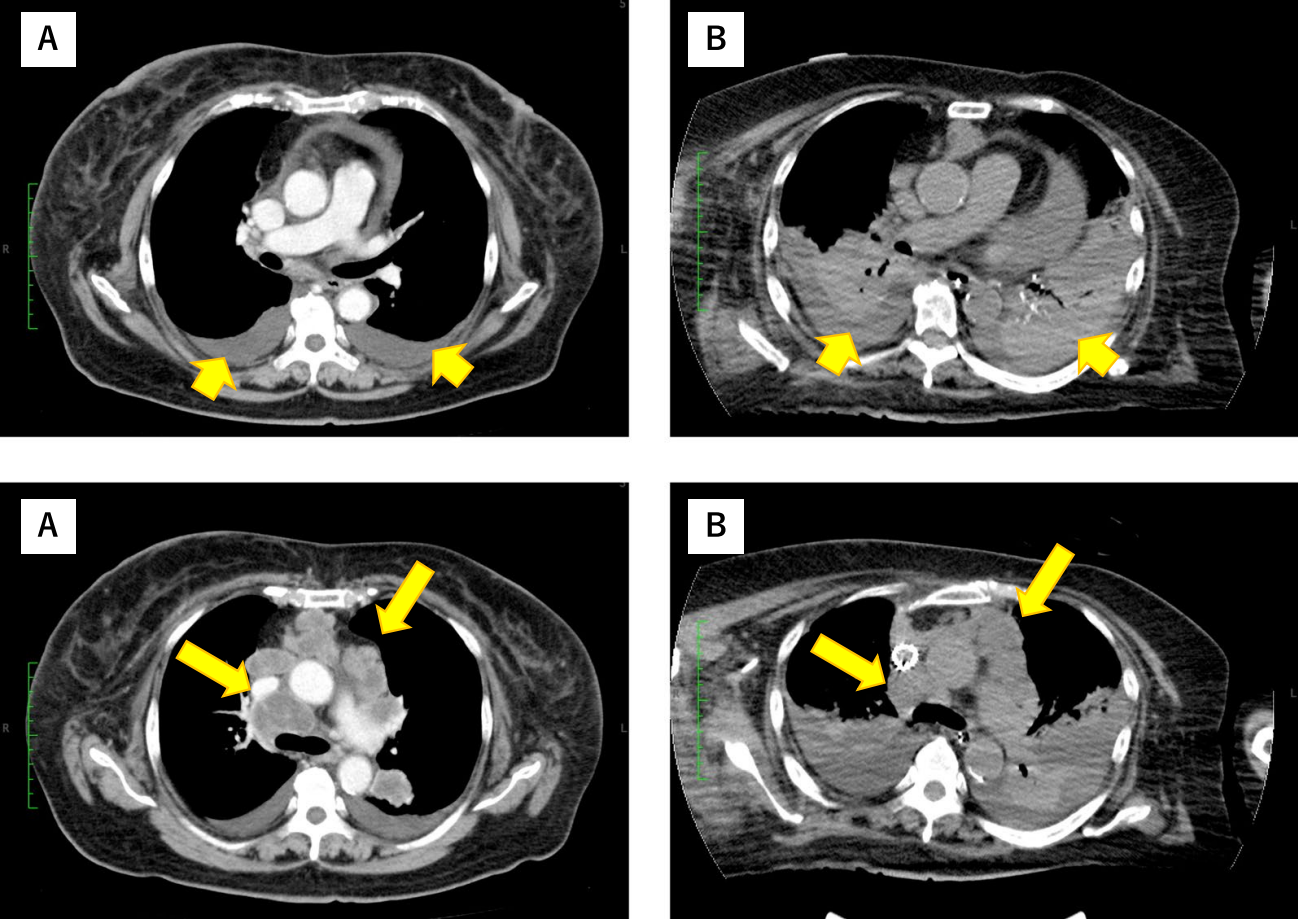
Chest computed tomography images before and after administering tepotinib. **a** Primary lung cancer in the left lower lobe, mediastinal lymph node metastasis, and bilateral pleural effusions before treatment. **b** Tumor progression in mediastinal lymph node metastasis (long arrow) and bilateral pleural effusion (short arrow)

*MET* mutations were confirmed in tumor specimens on day 21. Therefore, we decided to start the administration of MET inhibitor tepotinib (500 mg daily) through a stomach tube. However, her respiratory status failed to improve despite treatment, and right phrenic nerve paralysis was suspected because of tumor invasion. In addition, CT images on day 14 showed an increase in bilateral pleural effusion without any tumor shrinkage (Fig. 2). As it was difficult to improve the patient’s condition, tepotinib was discontinued. The patient received palliative care and died at the hospital on day 35.

### Case 3

A 50-year-old Japanese man presented with symptoms of chronic cough and dyspnea and was diagnosed with stage IV NSCLC in October 2020. CT images showed primary lung cancer in the right lower lobe with mediastinal lymph node metastasis. He was also diagnosed with right pleural effusion caused by cancer cells spreading to the pleura. He had never smoked and had no relevant medical history. Following transbronchial biopsy, adenocarcinoma was pathologically confirmed. The patient’s initial PS score was 0. The patient immediately underwent first-line chemotherapy consisting of carboplatin (area under the concentration–time curve 6, day 1), pemetrexed (500 mg/m^2^, day 1), and pembrolizumab (200 mg/body, day 1). On day 14 of the first cycle, the patient presented to the hospital with fever and dyspnea and was subsequently diagnosed with febrile neutropenia and acute pneumonia. In addition, the patient was suspected to have developed tumor progression, involving a primary lesion and pleural dissemination. The patient underwent endotracheal intubation because of worsening respiratory status, and immediately started receiving intravenous antibiotics.

**Fig. 3:**
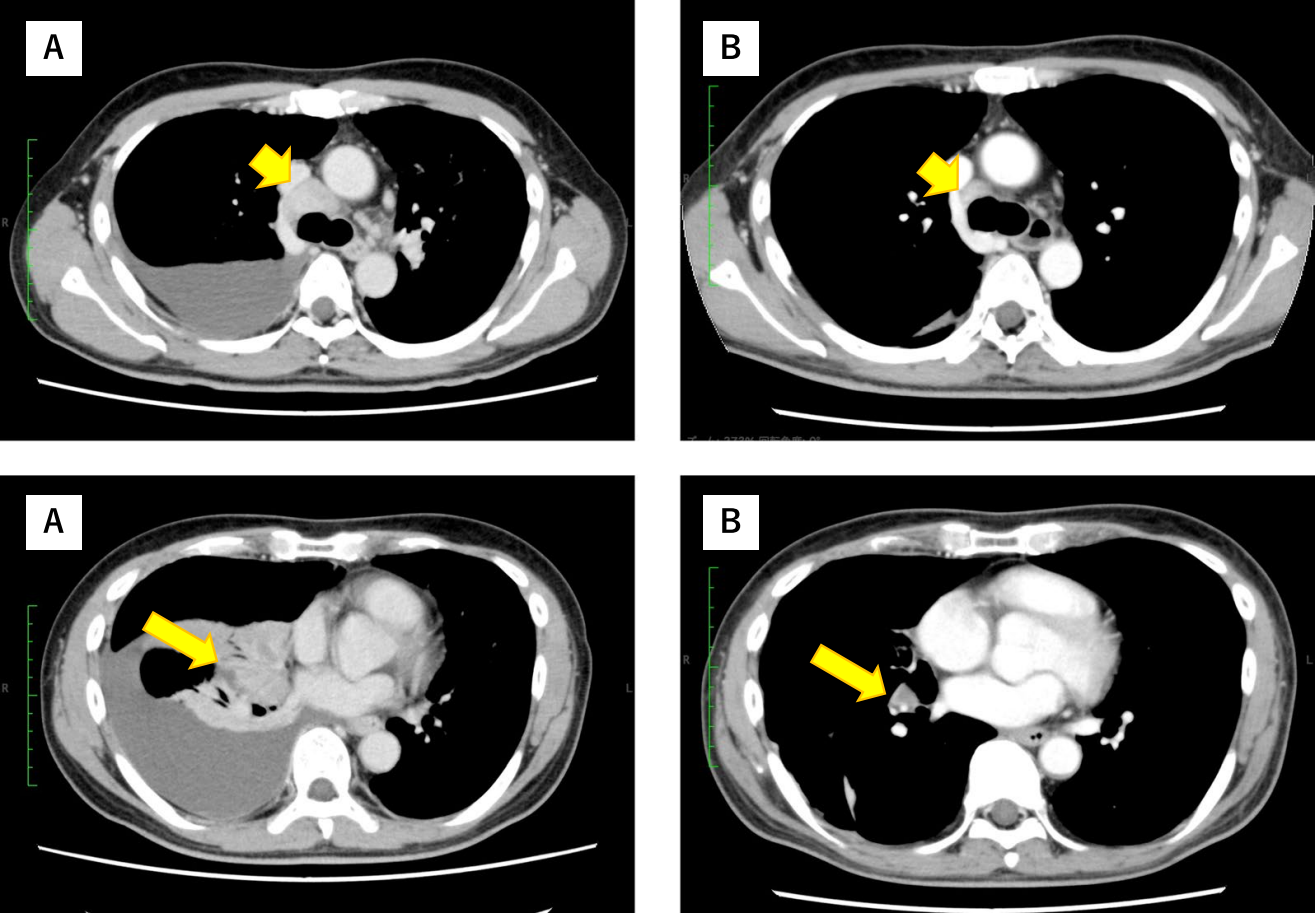
Chest computed tomography images before and after administering crizotinib. **a** Primary lung cancer in the right lower lobe, mediastinal lymph node metastasis, and right pleural effusion before treatment. **b** Significant tumor reduction in the primary tumor and (long arrow) mediastinal lymph node metastasis (short arrow). The right pleural effusion had disappeared after administering crizotinib

Genotype testing revealed an *ROS1* mutation; therefore, the patient was administered crizotinib (500 mg) daily through a stomach tube while on a mechanical ventilator. Grade 3 hepatotoxicity was observed on day 7 of the treatment. Crizotinib was discontinued until hepatotoxicity improved and, then, restarted at a reduced dose (400 mg) daily. A significant improvement in PS was noted within 2 weeks, with PS changing to 1. He underwent an initial evaluation of the therapeutic effects by CT scan on day 21 of the treatment. Significant shrinkage of the primary tumor and metastasis was detected, and the right pleural effusion had disappeared (Fig. 3). His general and respiratory condition improved, and he was discharged from the hospital on day 30 after recovery completion. Entrectinib replaced crizotinib as a measure against adverse events such as hepatotoxicity and taste disorders after discharge. However, in December 2021, he was treated with entrectinib without significant adverse events or tumor progression.

### Case 4

An 81-year-old Japanese woman was admitted to our hospital with cough and shortness of breath and was diagnosed with metastatic large-cell lung carcinoma in May 2021. CT findings revealed primary lung cancer; separate tumor nodules in the contralateral lobe; and multiple lymph node, bone, and adrenal metastases. The patient was admitted to the hospital because of hypoxemia, requiring oxygen administration. The patient’s initial PS was of 1, but her general and respiratory status worsened owing to cancer, resulting in a PS of 4 at treatment initiation.

**Fig. 4:**
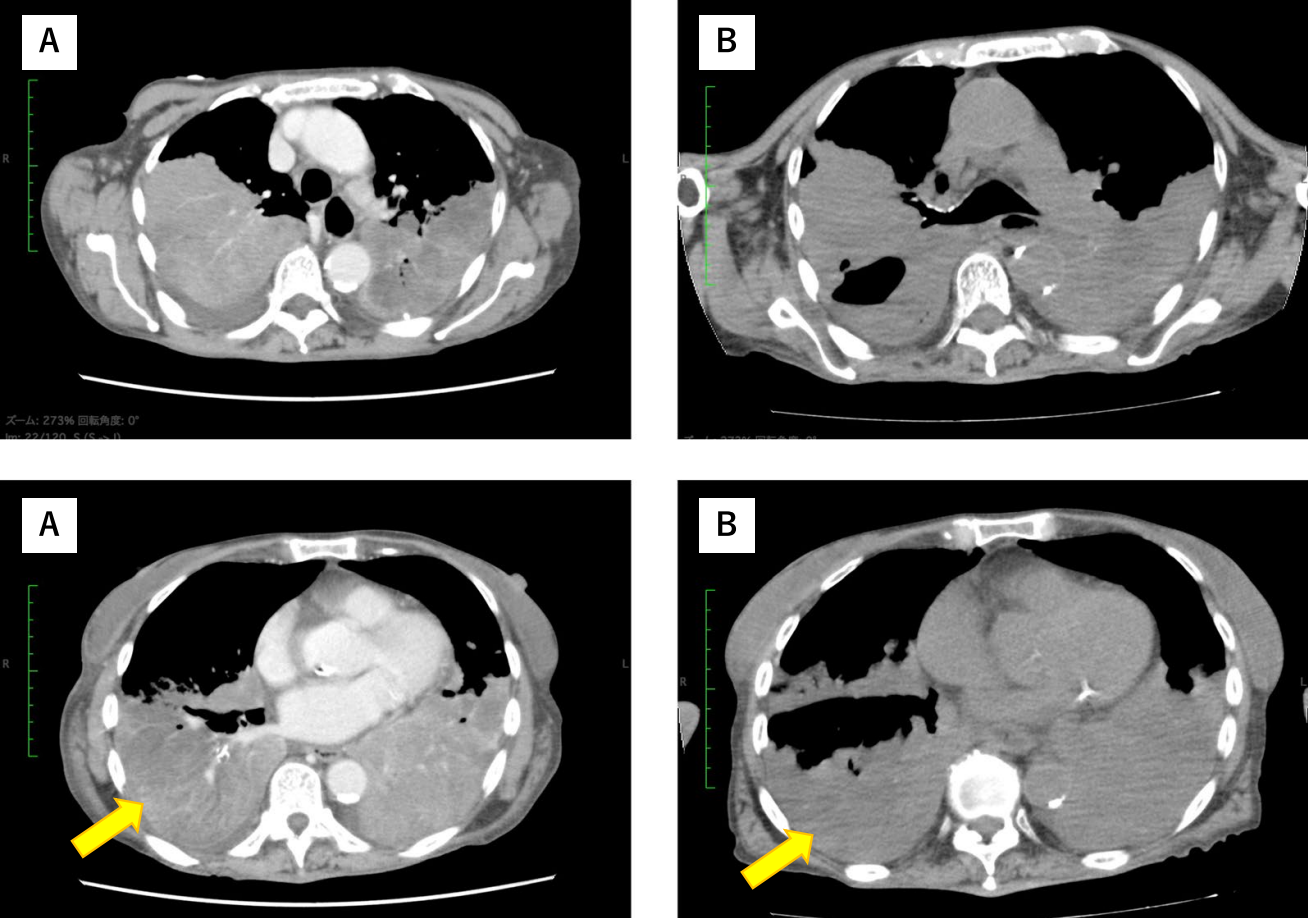
Chest computed tomography images before and after administering dabrafenib plus trametinib. The findings revealed **a** primary lung cancer and separate tumor nodules in the contralateral lobe before treatment and **b** slight tumor shrinkage in the right middle lobe (long arrow) without changes in the other lesions.

Genotype testing revealed a *BRAF*V600E mutation on day 21. The patient decided to undergo combination therapy with BRAF (dabrafenib) and mitogen-activated protein kinase inhibition (trametinib) [6]. She received a reduced dose of oral dabrafenib (200 mg) plus trametinib (1.5 mg) daily since she was older and had a poor PS. There were no significant adverse events after the initiation of therapy, and laboratory data were stable, including liver enzyme levels. Chest CT on treatment day 5 showed slight tumor shrinkage in the right middle lobe without changes in the other lesions (Fig. 4). However, on the 8th day of treatment, the patient developed nosocomial pneumonia, caused by *Staphylococcus aureus*. The patient did not go into septic shock, but her respiratory condition worsened despite antibiotic treatment, and she died of pneumonia on day 35.

## Discussion

We present a case series, in which four patients with NSCLC and PS 4 received TKIs for various alterations. To our knowledge, this is the first report to summarize the efficacy of TKIs in patients with PS 4. TKIs showed a significant effect in one patient, and his PS score improved to 0; however, no improvement in the PS scores of the other patients was observed, and they subsequently died in the hospital.

Chemotherapy is the standard treatment for patients with NSCLC with relapsed or advanced disease, as it prolongs survival compared to the best supportive care [15]. First, the patients scheduled to undergo chemotherapy are assessed for their PS by their doctors. The PS scale ranges from 0 to 4, with 0 denoting physical activity without restriction and 4 representing complete disability. Cytotoxic chemotherapy is indicated only for those with a good PS (0–2) before identifying driver alterations. In contrast, patients with a poor PS [3, 4] receive the best supportive care (BSC) [12, 13]. A comprehensive genomic analysis of NSCLC has identified several driver mutations and has guided the development of targeted TKIs in recent decades. Each TKI has shown a more effective response and fewer adverse events than those of the general cytotoxic chemotherapy. Therefore, it is recommended in the Japanese, NCCN and ESMO Clinical Practice Guidelines that tumors with driver alterations (e.g., *EGFR*, *ALK*, *ROS1*, *BRAF*V600E) be treated with front-line TKIs [12–14].

Additionally, previous studies have demonstrated that TKI therapy could have a favorable outcome and safety profile, even in patients with advanced NSCLC with a poor PS [9–11]. A phase-2 study suggested that patients with *EGFR* T790M mutation-positive advanced NSCLC with poor PS benefited from osimertinib treatment [11]; the PS improvement rate was > 70%, and nearly 90% of the patients improved from ≥ PS 3 at baseline to ≤ PS 2. Based on these results, the Japanese, NCCN and ESMO guidelines have recommended that each TKI be indicated even for diverse mutation-positive patients with a poor PS. Only *EGFR* and *ALK* TKIs showed significant improvement in patients with extremely poor PS. In contrast, there are no reports on the efficacy and safety of TKIs in patients with a poor PS harboring driver alterations of *EGFR*-uncommon, *ROS1*, *BRAF*V600E, *MET*, and *RET*. It is difficult to demonstrate TKI efficacy for patients with a poor PS and these driver alterations, because patients harboring these driver alterations are very rare. However, TKI treatment for these driver alterations is as effective as *EGFR* TKIs. Therefore, Japanese, NCCN and ESMO guidelines have recommended that TKIs of *EGFR*-uncommon, *ROS1*, *BRAF*V600E, *MET*, and *RET* are indicated for each driver mutation-positive patient with a poor PS.

In this case series, we analyzed driver gene alterations and TKI treatment for patients with NSCLC with a PS of 4 based on the above recommendations. One patient showed significant shrinkage in multiple lesions, and the PS score improved to 0 after TKI treatment. Two others showed slight shrinkage of the tumors but the PS score was not improved. Another patient showed tumor progression but the PS score was not improved. The three patients who could not establish an improvement in PS died in the hospital. All patients had a PS of 0–2 before being hospitalized, but three out of four patients were late-stage older individuals, aged ≥ 80 years. In addition, it took approximately 3 weeks for the patients to obtain the results of driver alteration analysis after hospitalization. They spent the entire time in bed, and their PS gradually worsened.

There are no upper age limits for the administration of TKIs in the guidelines. Furthermore, a previous study, which was conducted for patients with poor PS, did not perform a subgroup analysis of patients’ ages. Therefore, we usually proceed with the analysis of driver gene alterations and indication for TKIs, even for older patients and those with poor PS, in clinical practice.

**Fig. 5:**
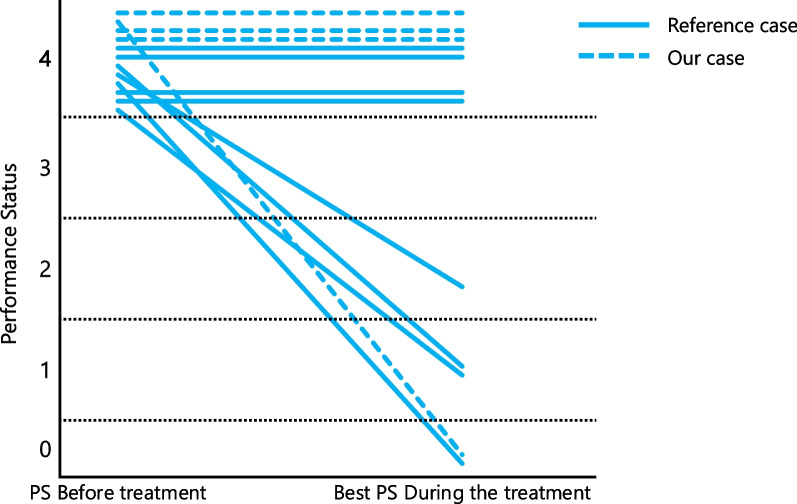
Change of the performance status of each patient during treatment. Each line shows the change of the performance status of a patient from baseline to the best status during the treatment. The solid line shows the case of reference, while the dotted line presents our cases

Table 1 summarizes several reports that described 12 cases of patients with PS 4 after TKI administration, including our cases [11, 16–21]. Additionally, Fig. 5 shows the change in PS for 12 cases during TKI treatment. Improved PS scores were observed in half of the patients. Almost all patients with improved PS were young or started TKI treatment early, owing to liquid biopsy. These results imply that younger age and early administration may be factors for PS improvement. Conversely, only one case reported that the PS of an older patient was significantly improved by TKI treatment over 3 weeks after hospitalization.

**Table 1:** Review of tyrosine kinase inhibitors for patients with non-small-cell lung cancer with a performance status of 4

References	Age	Sex	Type of driver alterations	TKI	Methods for the analysis of driver alterations	TKI response	PS change during TKI
Nakashima *et al.* [11]	78	F	*EGFR* exon 19 deletion	Osimertinib	Liquid	SD	4 → 1
Nakashima *et al.* [11]	71	M	*EGFR* L858R	Osimertinib	Liquid	PD	4 → 4
Nonagase *et al.* [16]	53	M	*EGFR* exon 19 deletion	Gefitinib	NR	PR	4 → 4
Matsuo *et al.* [17]	85	M	*ALK*	Crizotinib	Tissue	PR	4 → 0
Nishii *et al.* [18]	72	F	*EGFR* exon 19 deletion/T790M	Osimertinib	Liquid	CR	4 → 2
Tanaka *et al.* [19]	52	M	*ALK*	Alectinib	Tissue	PR	4 → 1
Wu *et al.* [20]	47	F	*ROS1*	Crizotinib	Tissue	SD	4 → 4
Xie *et al.* [21]	51	M	*EGFR* exon 19 deletion/T790M, *BRAF*V600E	Osimertinib and vemurafenib	Liquid	SD	4 → 1
Present case	89	F	*EGFR* L858R	Osimertinib	Tissue	SD	4 → 4
Present case	80	F	*MET*	Tepotinib	Tissue	PD	4 → 4
Present case	50	M	*ROS1*	Crizotinib	Tissue	PR	4 → 0
Present case	81	F	*BRAF*V600E	Dabrafenib plus trametinib	Tissue	SD	4 → 4

*ALK* anaplastic lymphoma kinase, *EGFR* epidermal growth factor receptor, *F* female, *NR* not reported, *NSCLC* non-small-cell lung cancer, *PD* progressive disease, *PR* partial response, *PS* performance status, *M* male, *ROS1* proto-oncogene receptor tyrosine kinase, *SD* stable disease, *TKI* tyrosine kinase inhibitor

Certainly, several TKIs have the potential to dramatically improve a patient’s condition, even if their PS is very poor, such as the so-called “Lazarus effect” [22]. However, it is imperative that we differentiate between the deterioration of PS owing to advanced age and instigated by malignant tumors. Previous studies have reported that older patients have worsened PS during hospitalization [23, 24], as it easily aggravates muscular strength and cognitive function, even though patients are in good general condition before admission. This hospitalization-associated disability occurs in more than one-third of patients aged > 70 years [25]. Additionally, worse general conditions cannot be reversed in more than half of older patients [26]. In our case series, most patients did not show improvement in PS after TKI administration. Continuous TKI treatment for older patients would be difficult due to aggravation of PS, even if the cancer responds to TKI treatment.

In contrast, patients with NSCLC can select BSC from the start when they do not want to undergo chemotherapy. In this case, they need not undergo invasive examinations or treatments, such as bronchoscopy, central intravenous catheters, or mechanical ventilation. In addition, these patients could spend time with their families at home if they wanted to. It has become available for indications of chemotherapy, especially TKI, even in older patients with a poor PS, because of the development of cancer treatment. A previous study reported that an improvement in the PS of patients with cancer led to an enhancement in their quality of life [27]. Namely, these patients might enjoy the rest of their lives with a good quality of life instead of spending the end of their lives in bed. However, some patients might spend different amounts of time in the terminal phase owing to the potential to receive chemotherapy. Regarding this point, the early involvement of palliative care, especially in older patients with NSCLC with a poor PS, would help guide the appropriateness of the use of TKIs in such patients.

Recently, several trials have evaluated the utility of circulating tumor DNA (ctDNA) genotyping, reporting that the screening duration of ctDNA genotyping is significantly shorter than that of tissue genotyping [28]. This utility may provide an early indication of TKI treatment without aggravation of patient PS. In fact, liquid biopsy was used in the patient’s improved PS with TKI therapy in previous case reports (Table 1).

## Conclusions

In this case series, we examined four cases of TKI usage in patients with NSCLC with a poor PS. One patient demonstrated an improvement in his general condition after TKI administration. However, despite receiving TKI treatment, three older patients, did not exhibit any improvement and ultimately passed away. The appropriateness of TKI treatment in patients with a PS of 4, particularly among older individuals, warrants further discussion. The potential for patients to endure an unwanted terminal phase due to invasive treatment cannot be overlooked. However, ctDNA genotyping, such as liquid biopsy, might initiate the treatment without worsening PS in the future. Our findings underscore the necessity for more comprehensive research in larger cohorts to conclusively assess the effectiveness of TKI treatment for patients with NSCLC with a poor PS.

## Data Availability

Not applicable.

## Abbreviations

ALK: Anaplastic lymphoma kinase
BSC: Best supportive care
CT: Computed tomography
ctDNA: Circulating tumor DNA
EGFR: Epidermal growth factor receptor
ESMO: European Society for Medical Oncology
NCCN: National Comprehensive Cancer Network
NSCLC: Non-small-cell lung cancer
PD: Progressive disease
PR: Partial response
PS: Performance status
ROS1: Proto-oncogene receptor tyrosine kinase
SD: Stable disease
TKIs: Tyrosine kinase inhibitors
